# Naa10 in development and disease

**DOI:** 10.18632/oncotarget.5867

**Published:** 2015-09-28

**Authors:** Line M. Myklebust, Svein I. Støve, Thomas Arnesen

**Affiliations:** Department of Molecular Biology, University of Bergen and Department of Surgery, Haukeland University Hospital, Norway

**Keywords:** Naa10, NatA, N-terminal acetylation, acetyltransferase, Ogden syndrome

Identification of causative mutations for rare genetic diseases has for long been of interest to medical geneticists. New developments within next generation sequencing have resulted in a huge increase in discovered pathogenic mutations. This is of great importance as it is the first step in the process of understanding the underlying mechanisms of different disorders. In 2011, such a sequencing study led to the identification of a point mutation in the N-terminal acetyltransferase Naa10 as the cause of a previously undescribed lethal disorder called Ogden Syndrome [1]. The *NAA10* gene was previously found to be overexpressed in different types of cancer, but its dysfunction had never before been shown to cause disease.

The vast majority of proteins undergo a broad range of chemical modifications either during or after their biosynthesis. These modifications increase the diversity of expressed proteins and are often crucial for their regulation and function. Protein Nα-terminal acetylation (Nt-acetylation) represents one such major modification affecting 80-90% of all soluble human proteins [2]. The N-terminal acetyltransferases (NATs) catalyze this reaction and transfer the acetyl moiety from acetyl-coenzyme A to the Nα-group of proteins' N-termini. In humans, six NAT enzymes (NatA-NatF) exist, acetylating defined sets of substrates [3]. Naa10 constitute the catalytic subunit of the NatA complex, the major NAT acetylating 40% of all cellular proteins [2].

**Figure 1:**
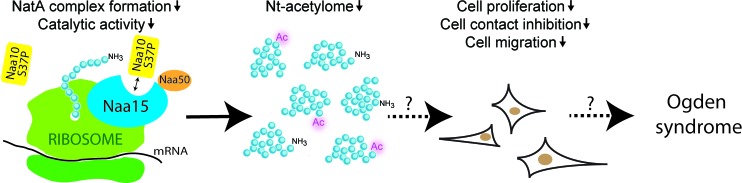
Effects of the *NAA10* p.S37P mutation in Ogden syndrome cells Cells expressing the Naa10 S37P variant have a reduced NatA complex formation and a reduced Naa10/NatA catalytic activity. The reduced catalytic activity causes a reduced Nt-acetylation of NatA substrates. Cells expressing the Naa10 S37P variant also have a reduced cell proliferation, a reduced cell contact inhibition and a reduced cell migration.

Ogden syndrome is an X-linked disorder characterized by severe global developmental delays, craniofacial anomalies, hypotonia, cardiac arrhythmia and eventual cardiomyopathy, resulting in mortality during infancy [1]. Our recent study highlights the molecular defects of the Naa10S37P variant causing the Ogden syndrome as well as the downstream cellular implications [4]. The mutant NatA complex displayed both an impaired peptide substrate binding and NAT activity (Figure 1) and furthermore, we observed a significantly reduced NatA complex formation. Our proteomic studies demonstrated a reduced Nt-acetylation level in both B-cells and fibroblasts derived from individuals with Ogden syndrome, while female carriers and wildtype family members had unchanged Nt-acetylation levels. This is the first time a human pathological condition has been linked to altered Nt-acetylation patterns. Interestingly, we observed a reduced Nt-acetylation only for a specific subset of the proteome matching the specificity of the NatA complex, thus supporting that NatA mediated acetylation is specifically perturbed *in vivo* in Ogden syndrome males. Ogden syndrome cells had a reduced growth rate and were less viable when cultured dispersed and stressed, but more metabolically active when kept in a dense culture. While wildtype cells entered the G0 phase, S37P cells continued to proliferate and showed a partial loss of cell-to-cell contact inhibition.

Naa10 is a key player in a variety of cellular pathways [3] and markers for these were investigated in Ogden syndrome cells. S37P cells revealed an increased expression of Retinoblastoma 1 (RB1), a known negative regulator of the cell cycle. Other pathways previously linked to Naa10 function such as β-catenin, Cyclin D, TSC2/mTOR/pS6K1, MYLK and β-PIX were not perturbed in S37P cells.

Several studies of female carriers of X-linked mutations have shown a skewed X-chromosome inactivation, a process occurring during early development and transmitted through subsequent mitosis favoring selection of the fit cell. In our study, all carriers of the *NAA10* p.S37P mutation were skewed toward the wild-type *NAA10* allele explaining why these females were healthy [4].

Naa10 is conserved from yeast to humans, and the critical role of Naa10 in normal development and disease was recently further demonstrated by *NAA10* knock-down studies in *Danio rerio* [5]. *NAA10* morphants displayed increased lethality, growth retardation and severe developmental abnormalities, and revealed that Naa10 is essential for early development and viability of zebrafish. This emphasizes the importance of a normal expression level of a functional NAT/Naa10.

In addition to the Ogden syndrome, further cases involving pathogenic *NAA10* mutations were recently presented. Lenz microphthalmia syndrome is caused by a splice site mutation c.471+2T>A in four affected males of the same family [6]. These presented with congenital bilateral anophthalmia, postnatal growth failure, hypotonia and skeletal anomalies. The affected males had mild to severe intellectual disability (ID) and delayed motor development. *De novo NAA10* mutations in the catalytic domain of Naa10 were found in males and females with intellectual disability, arrhythmia and developmental delays [7].

The variety of clinical manifestations in the patients described so far suggest that Naa10 mutants not only cause disease through one specific mechanism, but rather have pleiotropic effects, affecting different cellular functions. Some *NAA10* mutations might cause a general reduction of Nt-acetylation, and thereby cause disease due to loss of acetylation of key substrates that are important for control and regulation of physiological processes, while other mutations might cause disease due to non-catalytic effects mediated by Naa10. This highlights how further molecular and cellular studies of different *NAA10* mutations are vital in order to understand how these mutations are causing various human pathologies.
